# Dimensionless Parameters Define Criteria for Optimal Flow Velocity in Enhancing Chemotactic Response toward Residual Contaminants in Porous Media

**DOI:** 10.1021/acs.est.4c08491

**Published:** 2025-03-08

**Authors:** Beibei Gao, Roseanne M. Ford

**Affiliations:** Department of Chemical Engineering, University of Virginia, Charlottesville, Virginia 22903, United States

**Keywords:** bacterial chemotaxis, nonaqueous phase liquid (NAPL), optimal fluid velocity, Péclet number, time scale, microfluidics

## Abstract

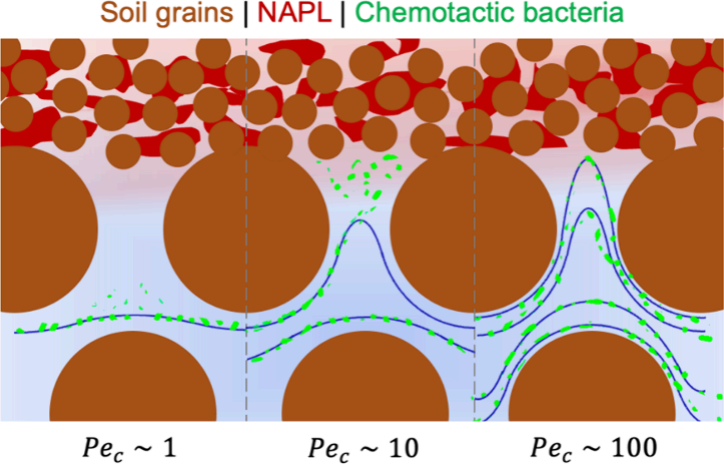

Chemotactic bacteria may overcome challenges posed by nonaqueous-phase liquid (NAPL) contaminants of low solubility in groundwater and limited bioavailability in tight pores by preferentially migrating to NAPL sources. We explored the transport of chemotactic bacteria to NAPL ganglia at varying pore water velocities in a dual-permeability microfluidic device and using computer-simulated solutions of transport equations. In our experiments, bacteria exhibited a chemotactic response toward NAPL ganglia at the junctures of low- and high-permeability regions (i.e., micropockets), and the extent of retention initially increased with velocity and then decreased at the highest velocity. A dimensional analysis revealed that maximum accumulations occurred at moderate values of the Péclet number  ∼10 in our system. We also found that accumulation dynamics in micropockets can be represented by a logistic equation incorporating convection and chemotaxis time scales  and , respectively. By analyzing seven literature studies on chemotaxis, we identified an exposure time scale to chemicals  that was useful for evaluating the chemotaxis efficiency. Our study provided unique insights into the effect of fluid flow on chemotaxis in porous media by demonstrating that increasing the fluid velocity to some extent can promote chemotaxis. The dimensionless parameters inform the design of efficient bioremediation strategies for contaminated porous media.

## Abstract 2

Chemotaxis can be enhanced by fluid flow rather than simply being suppressed. This work identified Péclet number criteria and relevant time scales to define optimal conditions with implications for bioremediation.

## Introduction

Remediation of nonaqueous phase liquids (or NAPLs) in subsurface environments remains challenging due to relatively low water solubility, mass transfer limitations, and multiphase flow conditions.^1,2^ The effectiveness of conventional groundwater extraction and injection technology is limited by subsurface heterogeneities where residual NAPLs are retained in low permeability strata that may leach contaminants into groundwater for decades.^2,3^ Bioremediation holds promise as an effective alternative to aid complete removal of NAPLs post pump-and-treat systems. Soil bacteria, such as **Pseudomonas putida** species, can detect the presence of hydrocarbons (e.g., toluene, benzene, and naphthalene)^4,5^ and alter their swimming behaviors in order to gather around chemical sources, which is termed chemotaxis. Although chemotaxis has been shown to enhance bacterial accumulation around NAPLs in laboratory studies,^6−9^ the impact of chemotaxis on bacterial transport in conditions more representative of natural subsurface environments still remains uncertain.

Bacteria living in soils are affected by the subsurface heterogeneity and fluid flow. Bacterial motility and chemotaxis have been studied extensively in microfluidic devices, which enable direct observations and precise control of microenvironments.^10^ In a flow-free microfluidic channel with chemoattractants, bacteria exhibit biased migration toward higher chemical concentrations.^11,12^ Fluid flow can interfere with bacterial swimming patterns, causing bacteria to become aligned with the flow direction.^13^ When a bacterial suspension passes through a porous microfluidic network with trapped NAPL droplets, fluid flow reduces bacterial retention near NAPLs.^14^ A microfluidic device with randomly distributed obstacles showed that bacterial dispersion in porous media was regulated by flow disorder.^15^ Subsurface environments, characterized by heterogeneous permeability (e.g., aquifers and aquitards) and flow regions (e.g., hydraulic shortcuts and bottlenecks), present complex challenges for understanding bacterial transport. Investigating bacterial chemotaxis in these heterogeneous porous media is crucial for optimizing bioremediation strategies.

Therefore, this work aimed to investigate the transport mechanism of chemotactic bacteria in a dual-permeability microfluidic device that mimics heterogeneous subsurface environments with fast preferential flow channels and low permeability barriers. NAPL ganglia were trapped in small pores in low permeable regions, while chemotactic bacteria were introduced into the highly permeable network at varying flow rates, and bacterial distributions were recorded by a microscope. A dimensional analysis study was conducted to investigate the fundamental transport processes of bacteria, integrating experimental observations with numerical simulations of bacterial transport. The findings of this work can be used to promote the efficiency of chemotaxis in cleaning up the contaminated subsurface.

## Materials and Methods

### Bacteria and Culture Conditions

**Pseudomonas putida** strain G7 (*Pp*G7),^16^ chemotactic to naphthalene, was used in this study. A bacterial overnight culture was prepared by following the protocol in Grimm and Harwood^16^ with modifications that were described in detail in Gao et al.^17^ Prior to use in subsequent experiments, bacterial motility was confirmed under oil immersion at 100× of a Zeiss microscope (F100/1.25 oil).

### Microfluidic Device

**Figure 1:**
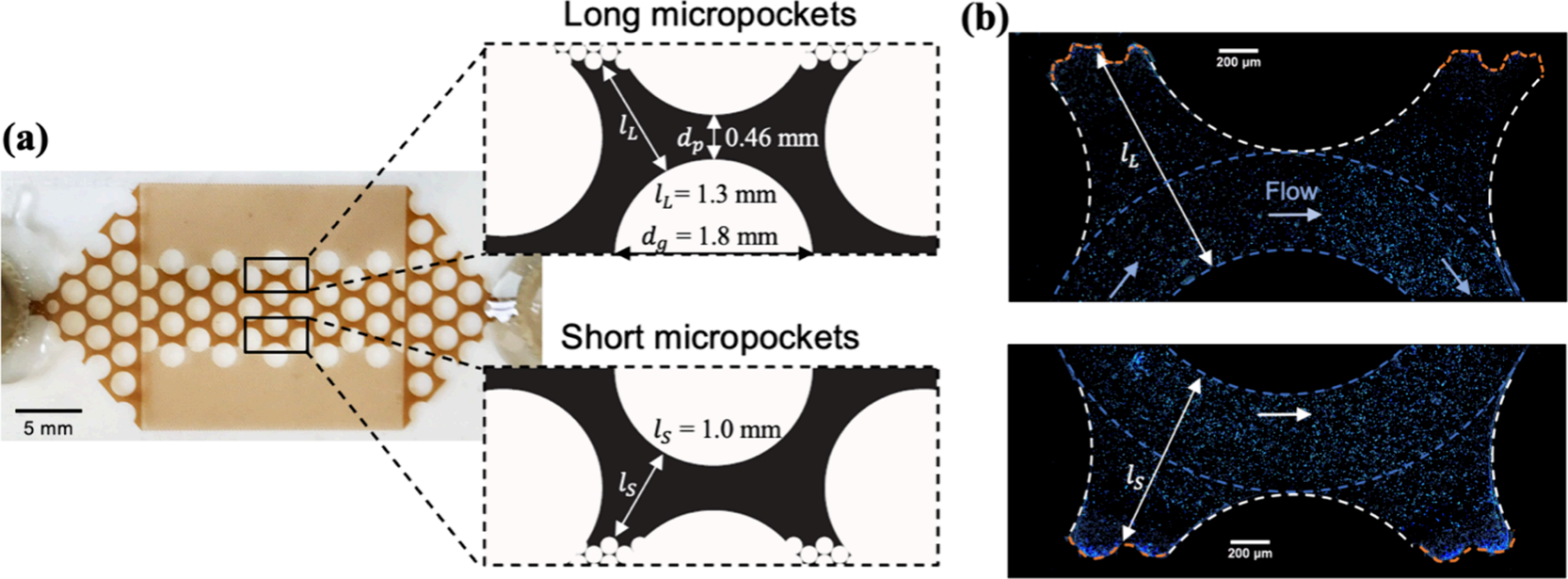
Dual-permeability microfluidic device used in this study. (a) Microfluidic device was saturated with an oil that is brown in color to visualize the porous network for demonstration purposes. The high-permeability area was sandwiched between low permeable areas, and the junctures were referred to as “micropockets” that had different lengths (*l*_*L*_ and *l*_*S*_), as shown in the enlarged view (pore space in black). (b) Images of micropockets and individual bacteria (dots) in chemotaxis experiments. NAPL-water interfaces at the end of the micropockets were labeled by an orange dashed line. The enclosed areas by dashed blue and white lines represent the main flow pathway and the region of interest for quantifying the influence of chemotaxis on bacterial distribution, respectively.

A dual-permeability microfluidic device made of glass by Wenhao Microfluidic Technology (Suzhou, China) was used in the bacterial experiments, as shown in Figure 1a. The grain diameter and pore throat diameter were 1.8 and 0.46 mm, respectively, for the high-permeability region in the micromodel center. For the low-permeability zones on both sides of the micromodel, the grain diameter and pore throat diameter were 0.12 and 0.045 mm, respectively. The microfluidic device has a 20 μm gap between the top and bottom of the chamber. The microfluidic chamber was initially saturated by a NAPL mixture containing 33 g of naphthalene per 1 L of 2,2,4,4,6,8,8-Heptamethylnonane (HMN). The large variation in pore dimensions between high- and low-permeability zones resulted in the majority of NAPL being flushed out of the center area leaving much of the pore space open to flow, as shown in Figure 1b. NAPL–water interfaces, especially at the edges of the highly permeable area (i.e., micropockets), were regions of interest in bacterial transport experiments. Micropockets at these junctions had different lengths from the pillar edge to low permeability areas, representing pores of different dimensions in soil environments. The naphthalene-HMN mixture is colorless, so a brown oil mixture was used in Figure 1 to aid visualization.

### Bacterial Transport Experiments

As the majority of residual NAPL was retained in low-permeability zones, the bacterial suspension flowed primarily through the central zone over the range of experimental fluid velocities, which varied from 0.2 to 56 m/d. Fluid velocity was calculated by tracking cells along the main flow path (between dashed blue lines in Figure 1b) using the TrackMate plugin in ImageJ.^18^ This velocity range was selected to represent both natural groundwater flow rates and convectional pump-and-treat fluid velocities commonly encountered in subsurface environments.^19−23^ Reynolds numbers (*Re*) were estimated to range from 0.001 to 0.319, using the pore throat size (0.46 mm) as the characteristic length, indicating that the fluid inertia was negligible. *Pp*G7 are chemotactic to naphthalene, and HMN is not known to elicit chemotaxis in *Pp*G7 or be toxic to bacteria.^24^ Bacterial distribution near NAPL-water interfaces, especially in the micropockets (junctures of high- and low-permeability zones), was monitored and imaged over a period of 60 min. During the observation period, we assumed that changes in bacterial density in pores were due to transport instead of proliferation or decay of bacteria. After each trial, the micromodel was washed with a flowing detergent foam under high pressure to remove oil blobs and bacteria.

### Data Acquisition and Processing

**Figure 2:**
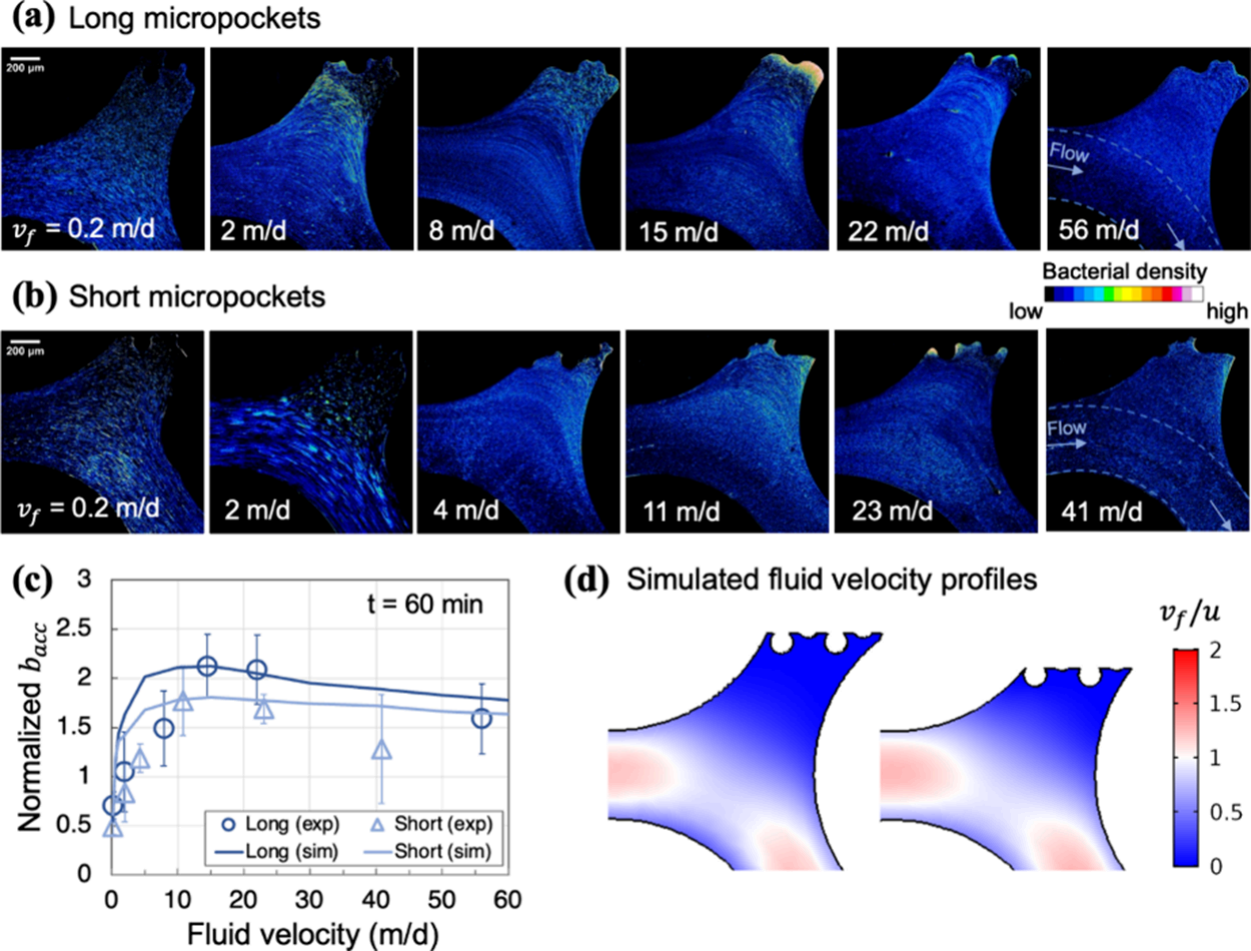
Bacterial distributions in micropockets for varying fluid velocities for 60 min. Panels (a) and (b), bacterial distributions in long and short micropockets, respectively, at junctures of high- and low-permeability areas in the micromodel. Top irregular boundaries of pore space were formed by NAPL-water interfaces. Arrows indicate the main fluid flow directions. (c) Averaged bacterial accumulation density across 10 micropockets was normalized to their density in preferential flow pathways (i.e., areas between dashed blue lines in Figure 1b) and plotted against fluid velocity (*v*_f_). Open circles and triangles represent experimental bacterial density, while dark and light lines represent simulated bacterial density at 60 min, in long and short micropockets, respectively. Error bars represent the standard deviation of bacterial densities across 10 micropocket replicates. (d) Simulated fluid velocity profiles in long (left-hand side) and short (right-hand side) micropockets. Pore velocity (*v*_f_) was normalized by an average velocity introduced at inlets (*u*).

Bacteria were imaged using a wide-field microscope (Olympus IX-70, FL) under a phase ring NO.1 and 10×/0.30 objective lens. 100 images were taken at each NAPL-water interface at a time interval of 50 ms every 15 min. Each 100-image stack was subtracted by an averaged image of itself in ImageJ to remove stuck cells on the ceiling or bottom of the chamber, and then the new stack was binarized to eliminate any background noise. Another averaged image was created from the binary stack (e.g., Figure 2a). Bacterial intensity in each pixel and pixel locations were imported to MATLAB. Computer simulations confirmed that bacterial density in the bulk fluid away from the NAPL surface (area outside dashed white lines in Figure 1b) was uniform. Therefore, the bacterial intensity in the enclosed area in Figure 1b was normalized by the average intensity in the bulk flow for a more consistent comparison among trials (e.g., normalized *b*_acc_ in Figure 2c).

### Mathematical Modeling and Simulation

Distributions of chemoattractant and chemotactic bacteria in the pore space were simulated by using diffusion-convection equations. We assumed no consumption of chemoattractant or growth or decay of bacteria during our experiments since no nutrient was provided and a bacterial suspension was continuously introduced into the system. The chemoattractant concentration in the aqueous phase was modeled by,
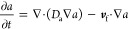
(1)where *a* is chemoattractant concentration in the aqueous phase (ML^–3^), *t* is time (T^–1^), *D*_a_ is the diffusion coefficient for the chemoattractant (L^2^ T^–1^), and *v*_f_ is the fluid velocity (LT^–1^). We assumed the chemoattractant concentration on the aqueous side of the NAPL–water interface was at its equilibrium concentration with the NAPL mixture (0.12 mol/m^3^) and that no chemical flux occurred in or out of the microfluidic wall boundaries. Initially, no chemoattractant was present in the highly permeable area.

For chemotactic bacteria, the directed movement toward the chemoattractant was accounted for by incorporating a chemotactic velocity^25,26^ into the convection term,
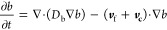
(2)
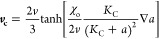
(3)where *b* is bacterial concentration in the aqueous phase (ML^–3^), *D*_b_ is the diffusion coefficient for bacteria (L^2^T^–1^), and *v*_c_ is the chemotactic velocity of a bacterial population (LT^–1^) which is a function of bacterial swimming speed *v* (LT^–1^), chemotactic sensitivity coefficient χ_o_ (L^2^T^–1^), and chemotactic receptor constant *K*_C_ (ML^–3^). We assumed no flux of bacteria at the NAPL–water interfaces or microfluidic walls. Initially, a bacterial suspension was introduced into the chamber at a fluid velocity, *v*_f_.

COMSOL Multiphysics version 5.6 was used to solve the differential equations for chemoattractant and bacteria in the identical high-permeability pore network, as shown in Figure 1. A screenshot of geometry and applied physics information is provided in Figure S1 (Supporting Information). Flow velocity (*v*_f_) ranged from 0.2 to 60 m/day, and other parameters used in our simulation are listed in Table S1. The two fitting parameters for bacteria (*D*_b_ and χ_o_) were obtained by matching bacterial density in micropockets (e.g., the area enclosed by the dashed white line in Figure 1b) with experimental data. Fitted values were within typical values as reported in the literature.^27,28^ A sensitivity analysis on *D*_b_ and χ_o_ can be found in the Supporting Information.

## Results and Discussion

### Bacterial Accumulation in Micropockets Depends on Pore Velocity

In bacterial transport experiments, chemotactic bacteria *Pp*G7 were introduced into the micromodel chamber in the presence of residual NAPL at varying fluid velocities from 0.20 to 56 m/d, which covers the range of natural groundwater and pump-and-treat fluid velocities in subsurface environments.^19−23^Figure 2a,b shows bacterial distributions at 60 min near NAPL surfaces in pores with different lengths as measured along the direction perpendicular to flow, referred to as long and short micropockets, respectively. Our previous work^17^ has shown that micropockets with NAPL sources were ideal locations for chemotactic bacterial accumulation. These regions were characterized by quiescent fluid conditions compared to main flow pathways (Figure S4, Supporting Information). Our previous work^17^ also proved that this accumulation was driven by chemotactic responses to persistent NAPL gradients in micropockets, as nonchemotactic bacteria, used as an experimental control for chemotaxis and driven by diffusion only, distributed uniformly within the micropocket. Building on this knowledge, in this investigation, we compared accumulation in micropockets at various flow velocities to experimental measurements (Figure 2c). As pore water velocities increased, both types of micropockets exhibited greater bacterial intensity near NAPL-water interfaces up to a point, and then intensity faded at the greatest velocities. The optimal fluid velocity yielded the maximum bacterial accumulation in micropockets. Fewer bacteria accumulated in short micropockets compared to those in long micropockets, which may result from relatively greater convection closer to NAPL surfaces in short micropockets, as shown by simulated pore velocity profiles in Figures 2d and S3.

### Optimal Fluid Velocity for Bacterial Chemotaxis

**Figure 3:**
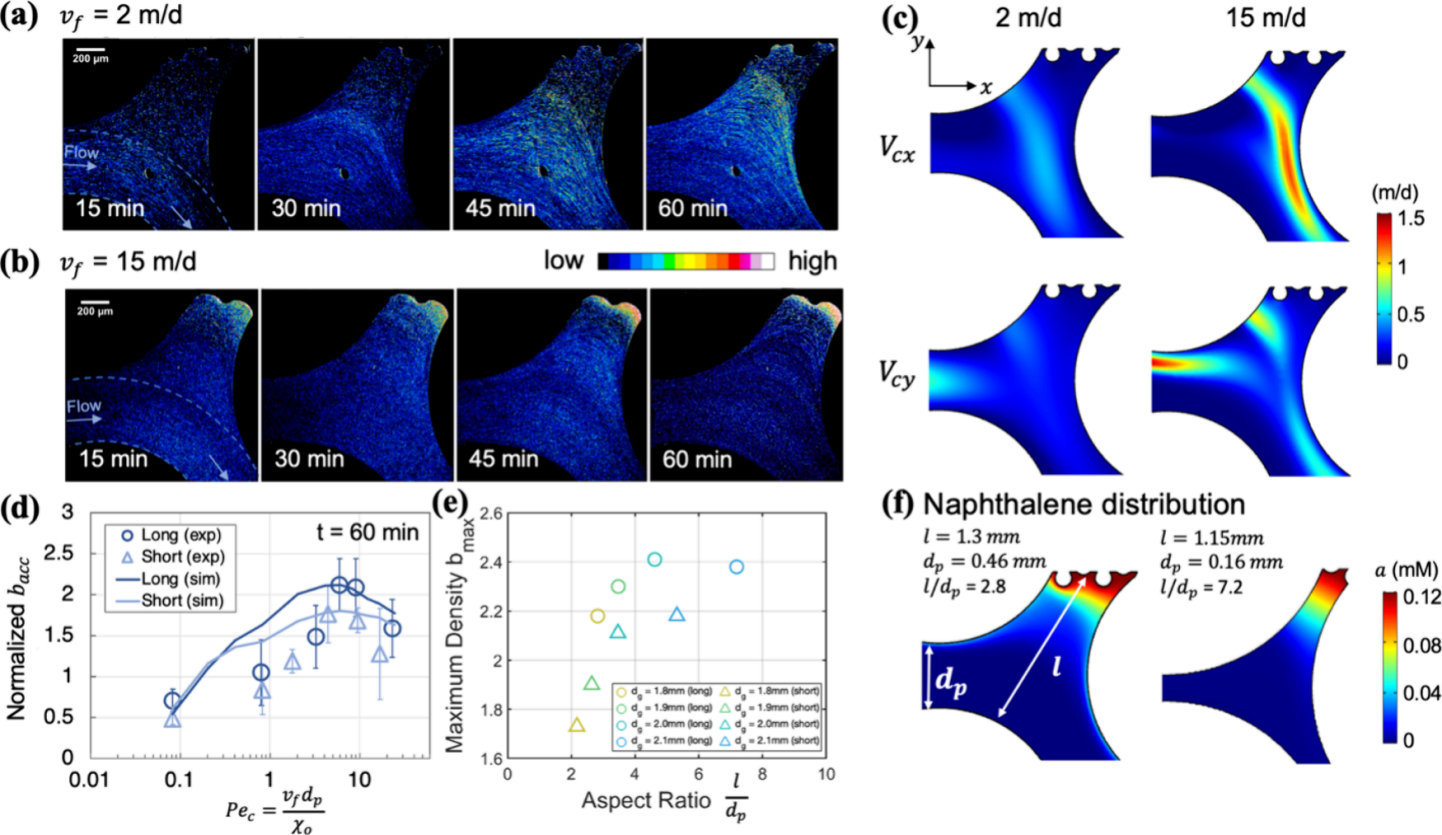
Fluid flow paths entering micropockets carried bacteria toward NAPL sources. Images of bacterial distributions in two individual long micropockets at different time points for average fluid velocities of (a) 2 and (b) 15 m/d. Arrows indicate the main fluid flow directions. (c) Chemotactic movement of bacteria in response to chemoattractant gradients was quantified using the chemotactic velocity *V*_c_. Simulated chemotactic velocities along *x* (*V*_c*x*_) and *y* (*V*_c*y*_) directions were compared and visualized at 2 and 15 m/d within long micropockets. (d) Simulated bacterial accumulation densities (*b*_acc_), averaged across all 10 micropockets, were plotted against a Peclet number  to help illustrate the balance between convection and chemotaxis on bacterial transport. At *Pe*_c_ approaching 10, bacterial accumulation achieved maximum densities in the micropockets. (e) Maximum bacterial densities (*b*_max_) obtained from simulations with four different geometries were plotted against pore aspect ratio , where *l* is pore length and *d*_p_ is pore throat width. (f) Visualization of simulated naphthalene concentrations in micropockets with the smallest and largest aspect ratios of 2.8 and 7.2 in our simulations.

The influence of fluid velocity on bacterial chemotaxis in porous media was more complex than simply hampering chemotactic response as reported in homogeneous liquid studies.^13,29^Figure 3a,b shows temporal-spatial changes in bacterial distribution near NAPL-water interfaces at two fluid velocities, 2 and 15 m/d. At 2 m/d, a bright band of bacteria at higher population density traveled toward a NAPL surface over the 60 min observation period. When fluid velocity was increased to 15 m/d, bacteria started accumulating near the NAPL surface in the first 15 min. Computer simulations confirmed that chemotactic velocity was greater at 15 m/d than that at 2 m/d (Figure 3c) in both the *x* and *y* directions. The vicinity of the NAPL–water interface was characterized by quiescent flow conditions and persistent chemical gradients. As the flow rate increased from 2 to 15 m/d, fluid flow paths advanced further into the micropocket, creating steeper chemical gradients, which not only brought bacteria closer to NAPL sources but also provoked a stronger chemotactic response, or chemotactic velocity (eq (3)). Therefore, bacteria accumulated more rapidly and extensively at 15 m/day compared to 2 m/day, as shown in Figures 2 and 3a,b.

In mass transfer, the Peclet number quantifies the ratio of convection to diffusion-driven transport. Similarly, bacterial transport in pores is influenced by convection and chemotaxis, which is driven by chemical gradients. To quantify their interplay, we defined a Peclet number for chemotactic bacteria, , to compare the relative contributions of pore-scale convection and chemotaxis, as shown in Figure 3d. Larger *Pe*_c_ values represent a regime where convective flushing dominates, while *Pe*_c_ < 1 corresponds to chemotaxis as the primary driver. However, as shown in Figure 3a,b (with *Pe*_c_ < 1), chemotaxis-dominant conditions may not always lead to significant bacterial accumulation. Our experiments suggest that *Pe*_c_ values between 5 and 10 represent an optimal range where convection and chemotaxis synergistically enhance bacterial migration toward chemoattractants in micropockets. Analogous to how the Reynolds number distinguishes laminar and turbulent flow regimes, *Pe*_c_ could be an insightful metric for identifying conditions that strike an optimal balance between convection and chemotaxis, maximizing bacterial densities in micropockets without the need to solve complex equations. Further investigations are needed to correlate optimal *Pe*_c_ values across different bacterial species, pore geometries, and chemical gradients.

At the optimal fluid velocity (*v*_m_), micropockets reached their maximum capacity for bacterial accumulation per unit area (*b*_max_). In our experiments, the maximum bacterial density *b*_max_ in long micropockets was about 26% higher than in short micropockets, suggesting that the micropocket shape played a significant role in bacterial accumulation. We quantified the micropocket shape using the aspect ratio *l*/*d*_p_. Across the four porous media in Figure 3d, an increase in grain diameter from 1.8 to 2.1 mm led to an increase in the aspect ratio (*l*/*d*_p_) from 2.8 to 7.2 for long micropockets and from 2.2 to 5.3 for short micropockets, as shown in Figure 3f. Simulation results suggested that longer and narrower pores, characterized by higher *l*/*d*_p_ values, supported greater bacterial accumulation densities. This finding provides new insights into the remediation of contaminants in narrow and elongated pores, such as those in compact subsoil layers, which are less accessible for conventional pump-and-treat flushing and require high energy for contaminant removal. Our study demonstrates that in elongated pores, bacterial density can increase even at lower fluid velocities, as chemotaxis allows bacteria to migrate the remaining distance toward contaminants. Optimizing fluid conditions for a given set of pore dimensions could help overcome the challenges of limited contaminant access in these environments by leveraging the targeted flushing of chemotactic bacteria.

### Chemotaxis and Convection Time scales

**Figure 4:**
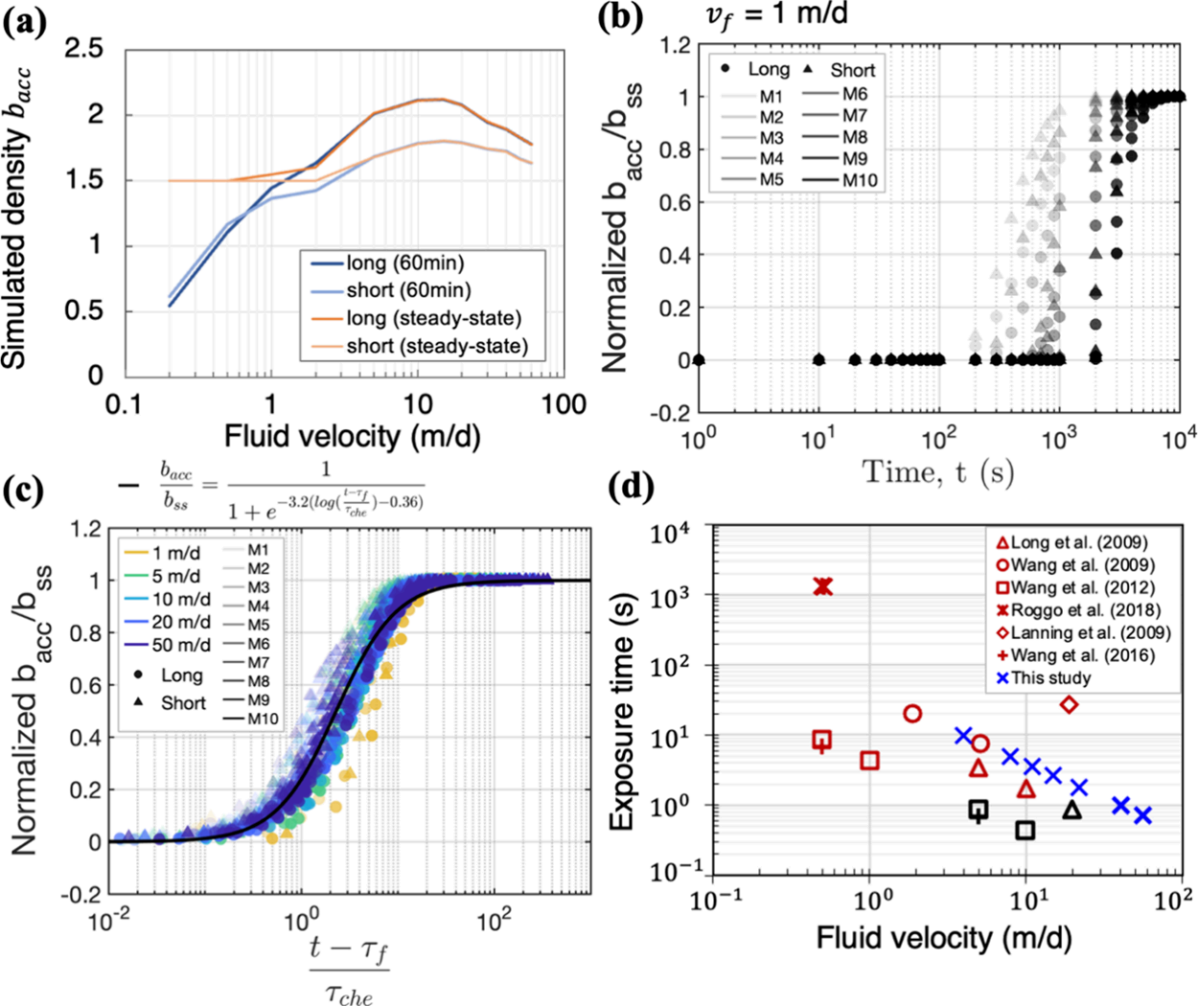
(a) Simulated bacterial densities averaged across 10 long and short micropockets at 60 min and under steady state conditions. Temporal change in simulated bacterial density within 10 long (circles) and short (triangles) micropockets at 1 m/day (b) and varying flow velocities (c) after scaling. The ten micropockets on each edge of the high-permeability region are labeled M1 (lightest shade) to M10 (darkest shade), representing their order from the bacterial suspension inlet along the flow direction to the exit. Bacterial density was scaled by its steady state value (*b*_ss_) for comparison. In (c), time was scaled by characteristic time scales for flow  and chemotaxis , where *L*_i_ is the micropocket distance from the entrance and *l*_*a*_ is the naphthalene gradient length. Solid black line is a logistic fitting with a *R*^2^ = 0.98. (d) Exposure time to chemicals (τ_exp_) estimated for experimental systems in this work (blue crosses) and the published literature was plotted against fluid velocity (*v*_f_). Chemotactic and nonchemotactic outcomes from the literature were labeled in red and black, respectively.

Simulations show that steady-state bacterial accumulation densities for flow velocities *v*_f_ < 2 m/d were higher than the densities predicted at 60 min (Figure 4a). Therefore, we note that at lower fluid velocities some micropockets did not reach a steady state at 60 min. Figure 4b illustrates the temporal evolution of bacterial densities in ten long (circles) and short (triangles) micropockets at 1 m/d. Micropockets are shaded to indicate their distance from the flow inlet with lighter shades (e.g., M1) representing micropockets closer to the entrance and darker shades (e.g., M10) representing those farther away. Simulations show that bacteria accumulated earlier in the first micropocket (M1) than in the tenth micropocket (M10), and earlier in short micropockets than in long ones. We found that the time required for bacterial accumulation in micropockets to reach a steady state under varying flow velocities could be estimated using two time scales: the convection time scale  and the chemotaxis time scale , as shown in Figure 4c. Here, τ_f_ represents the time scale needed for bacteria to be delivered to micropockets by convection, where *L*_i_ is the distance from the entrance to each micropocket. The locations of M1–M10 locations and their distance *L*_i_ were detailed in Figure S4c (Supporting Information). Subtracting τ_f_ largely eliminated the discrepancies in the bacteria arrival times among micropockets at different distances. Bacterial accumulation in micropockets is driven by chemotaxis in response to naphthalene gradients *l*_a_, which were estimated from simulations (Figure S5, Supporting Information). The chemotaxis time scale (τ_che_), inspired by the Einstein–Smoluchowski formula for Brownian motion,^30^ predicts the accumulation time scale based on the naphthalene gradient length *l*_*a*_ and chemotactic response χ_o_. τ_che_ captures the differences in bacterial accumulation between long and short micropockets located at the same distance from the flow entrance. Bacterial transport dynamics can be effectively described by these two time scales, τ_f_ and τ_che_, highlighting that directed migration in porous media can be determined by two key processes: convection that delivers bacteria to contaminated sites and chemotaxis that is driven by pore-scale chemical gradients. It is worth noticing that the unified curve in Figure 4c follows a logistic equation,^31^
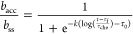
(4)with a logistic growth rate *k* = 3.2, sigmoid point τ_0_ = 0.36, and a fitting score of *R*^2^ = 0.98. The logistic equation describes a target change in an environment with limiting factors, such as analyte variation in a chromatographic system.^32^ In Figure 4c, *k* controls the steepness of the curve, indicating accumulation kinetics such that a higher *k* value suggests less mass transfer resistance or stronger chemotaxis. The midpoint τ_0_ corresponds to the maximum accumulation rate, after which accumulation slows until reaching the micropocket maximum capacity. The τ_0_ value depends on pore geometry, fluid disturbance, and chemotaxis strength, as it is determined by time scales τ_f_ and τ_che_. By comparing the midpoint and steepness of the logistic curve, we can gain valuable insights into bacterial interactions with porous media, allowing us to optimize conditions for better remediation efficiency. The fitted values for *k* and τ_0_ enable us to estimate the time required to reach the maximum accumulation capacity of micropockets at a given flow velocity as well as calculate the range of fluid velocities needed to meet the time requirement. In applications to real-world scenarios, smaller-scale lab experiments and simulations that mimic critical features of the system of interest would be performed to obtain these parameters for the logistic model. Analogous to an empirical correlation, the logistic model would allow one to scale up results by leveraging dimensional analysis to reduce the need for extensive experimentation in larger-scale systems of interest.

### Exposure and Response Time scales

In Figure 2c, bacterial accumulation in micropockets decreased as the fluid velocity increased beyond 20 m/d. Simulated results predicted greater accumulations than that of experiments at velocities faster than 40 m/d. Chemotaxis might not directly depend on flow rates, but rather on whether exposure is sufficient for bacteria to undergo internal changes that enable a chemotactic response. Chemotaxis is mediated internally by biochemical pathways with intrinsic time scales from seconds to minutes^33^ during which cells continuously sample their chemical environment and induce subsequent adjustments. **Pseudomonas putida** change their swimming direction once every two seconds on average^34^ and need several runs to exhibit biased migration toward chemical sources.^35^ In our study, the time scale of bacterial exposure to chemical gradients was estimated to be , where the pore throat length *d*_p_ was 460 μm (Figure 1a) and plotted against fluid velocity (*v*_f_) in Figure 4d (cross blue symbols). Figure 4d also summarized bacterial studies from the literature where bacteria moved through pores of various sizes across a wide range of flow rates,^14,29,36−39^ with red symbols indicating chemotactic response and black symbols showing no chemotactic response. Lanning et al.^39^ observed chemotaxis at 19 m/d where a bacterial suspension was mixed with chemicals immediately upon entering the horizontal channel. In contrast, in Long et al.,^36^ bacteria were unable to respond at a similar flow rate of 20 m/d when chemical gradients were limited to within the pore space. When the exposure time scale to chemicals was in proximity to or less than the bacterial response time (e.g., 2s for **Pseudomonas putida**), we expected that bacteria in micropockets to exhibit weak or no chemotaxis, as seen with the black symbols in Figure 4d and when *v*_f_ > 40 m/d in our experiments. The weaker response was quantified by smaller χ_o_ values in simulations, which yielded a closer alignment with experimental data (Figure S7 in the Supporting Information). The exposure time scale τ_exp_ closely approximated the bacterial residence time at the chemical gradient boundary within micropockets, as detailed in Figure S8 of the Supporting Information.

Mathematical approaches to modeling the chemotaxis of a bacterial population implicitly assumed that the chemotactic response was instantaneous.^40^ Ignoring the physiological limit of bacterial response time possibly caused the greater accumulation from simulation predictions in Figure 2c when *v*_f_ was higher than 40 m/d. By evaluating response time in bacteria, we can estimate the conditions under which we need to account for the finite time of individual bacteria to sense and respond to chemical gradients in predicting bacterial chemotaxis from conventional advection-dispersion equations. Therefore, by comparing the exposure time scale to the response time scale, we can predict the scenarios for which chemotaxis is expected to be significant. This approach provides a useful predictive framework, especially for porous media with a homogeneous pore space. Future work should aim to refine this framework by incorporating additional complexities, such as heterogeneities in pore sizes and local flow fields. These factors might significantly alter exposure and response dynamics. Incorporating these considerations into numerical models can enhance our ability to predict bacterial behaviors in more complex porous media.

### Relevance of Pore-Scale Behavior to In Situ Bioremediation

The bioavailability of contaminants is one of the limiting factors in the bioremediation of NAPLs.^2,41^ The microfluidic device used in this study was specifically designed to simulate heterogeneous layers with high (e.g., sand) and low (e.g., clay) permeability zones to address the challenge of residual contamination in low-permeability regions that are difficult to reach with pump-and-treat methods. In porous media, bacterial access to contaminants often relies on chemotactic migration through narrow pore throats between soil particles. This study highlights how fluid flow influences bacterial motility and chemotaxis, revealing that convective flow patterns can aid bacterial accumulation by carrying cells closer to NAPL sources trapped in low-permeability regions and creating steeper chemical gradients that enhance chemotactic responses. Notably, we identified an optimal range of flow velocities for the 2D micromodel used in this study, defined by a Peclet number (), where bacterial accumulation was maximized through a balance of convective mixing, flushing, and chemotaxis (*Pe*_c_∼ 10). In heterogeneous field environments, local variations in flow velocity could create microenvironments that either facilitate or hinder chemotactic bacterial accumulation. By modification of flow conditions as guided by *Pe*_c_, strategies such as bioaugmentation can be optimized to enhance bacterial retention at contaminated sites, particularly in low permeable layers. For instance, results from this study suggest that compact soils with narrower and longer pores (e.g., clay) may be expected to retain more bacteria under favorable flow conditions, improving access to residual NAPL in dead-end micropockets. Additionally, we used a logistic equation  to demonstrate bacterial accumulation kinetics in micropockets with the inclusion of convection and chemotaxis time scales, τ_f_ and τ_che_. This logistic equation related bacterial accumulation rates to pore dimensions, fluid velocities, and bacterial chemotaxis, providing a framework to guide pump-and-treat or other mixing operations to maximize bacterial efficiency.

At very high flow velocities, we observed weakened chemotaxis due to an inadequate exposure time for bacteria to sense and respond to chemical gradients. While our current modeling approach helps identify general trends in bacterial retention, it may overestimate bacterial accumulation in high-velocity regions. This outcome underscores the need for improved numerical models that incorporate bacterial response times and pore-scale heterogeneities. For practical field applications, integrating predictive models with real-time monitoring tools, such as tracer tests, qPCR, and direct imaging in pilot studies, can refine strategies and track bioremediation progress more effectively. By the development of tools to bridge laboratory findings with field-scale implementation, this work offers actionable insights to enhance bioremediation in complex subsurface environments.

Microfluidic devices provide controlled environments for systematic evaluation of key parameters, making them valuable for studying bacterial transport in subsurface systems. In this work, we developed a heterogeneous dual-permeability microchannel to replicate contaminated hotspots and preferential-flow pathways. Real-time visualization through microscopy offered new insights into bacterial movement and interactions with chemoattractants, fluids, and pore structures. The small scale of microfluidics reduces resource requirements and ensures reproducibility, but there are limitations. Their two-dimensional structure makes it challenging to replicate the three-dimensional complexity of natural porous media. Wall effects, material interactions, and nutrient supply constraints also pose challenges. In our experiments, for example, bacterial motility decreased after one hour due to nutrient depletion. Despite these constraints, microfluidics remains a powerful tool for studying mechanisms at smaller scales, providing valuable insights into informing in situ bioremediation strategies.

## Supporting Information Available

Simulation parameters and settings in COMSOL Multiphysics, along with details on parametric analysis, shear rate, naphthalene distribution, and bacterial residence time in micropockets (PDF)
