# Supplementary material for: Dimensionless Parameters Define Criteria for Optimal Flow Velocity in Enhancing Chemotactic Response toward Residual Contaminants in Porous Media
Source: Environ Sci Technol. 2025 Mar 8;59(10):5080–7. doi: 10.1021/acs.est.4c08491 (PMC11924221; doi:10.1021/acs.est.4c08491)
Supplement: Supplementary file 1 — es4c08491_si_001.pdf [file es4c08491_si_001.pdf]

SUPPORTING INFORMATION

Dimensionless parameters define criteria for optimal flow velocity in enhancing chemotactic response toward residual contaminants in porous media

*Beibei Gao and Roseanne M. Ford**

Department of Chemical Engineering, University of Virginia, Charlottesville, Virginia 22903, United States

*Corresponding authors. Roseanne M. Ford, phone: (+1) 434-924-6283; e-mail: rmf3f@virginia.edu. Mailing address: Department of Chemical Engineering, University of Virginia, 385 McCormick Rd, Charlottesville, VA 22903.

NUMBER OF PAGES: 13

NUMBER OF FIGURES: 8

NUMBER OF TABLES: 3

Parameters and Settings in COMSOL Multiphysics

Differential equations for chemoattractant and bacteria were solved using COMSOL Multiphysics software version 5.6 Transport of Diluted Species module. Figure S1 shows the implementation of our model into COMSOL and Table S1 lists parameters used in simulation.

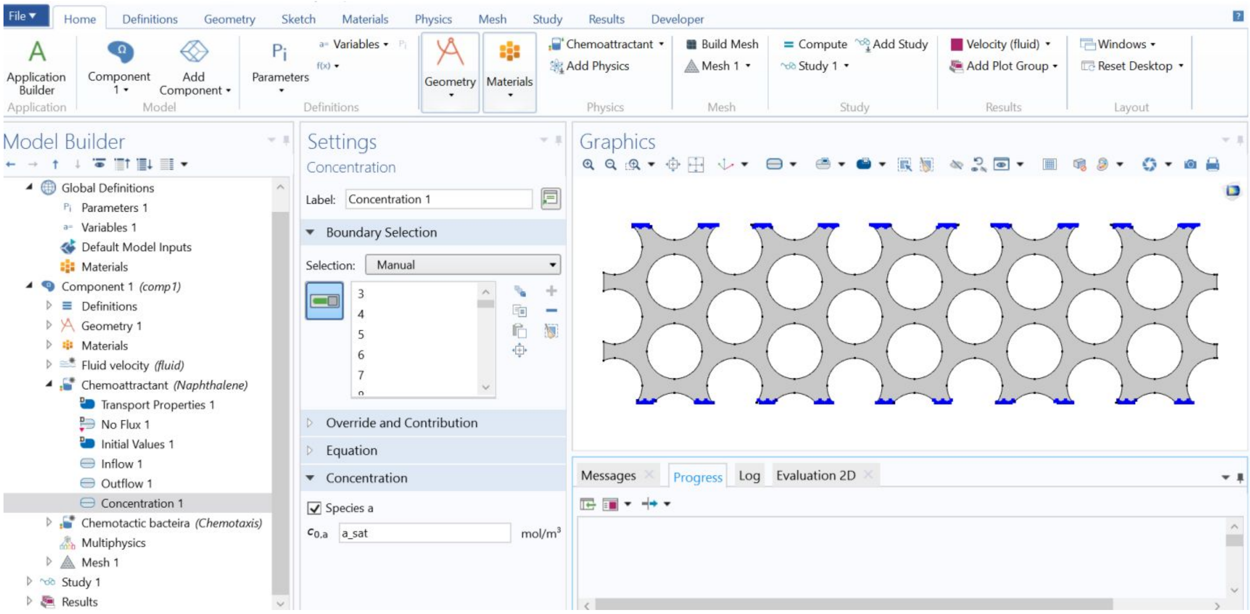

Figure S1. A screenshot of COMSOL Multiphysics Interface showing the geometry and physics used in our simulation. Blue lines in Graphics represent NAPL-water interfaces at the juncture of high- and low-permeability regions.

Table S1. Parameters used in chemoattractant and chemotactic bacteria simulation.

	Parameters	Values
Naphthalene	Diffusion coefficient, D_a (cm^2/s)	7.5×10^{-6}
	Solubility in water, a_{sat} (mM)	0.12
Bacteria	Diffusion coefficient, D_b (cm^2/s)	3.2×10^{-6} (fitted)

	Chemotactic sensitivity coefficient, χ_o (cm^2/s)	13 × 10^{-5} (fitted)
	Chemotaxis receptor constant, K_C (mM)	0.016 [a]
	Initial normalized concentration, b_0 (mM)	1
	swimming speed, v (um/s)	49 [a]

[a] Parameter values were obtained from Marx and Aitken[1].

Sensitivity Analysis of D_b and χ_o

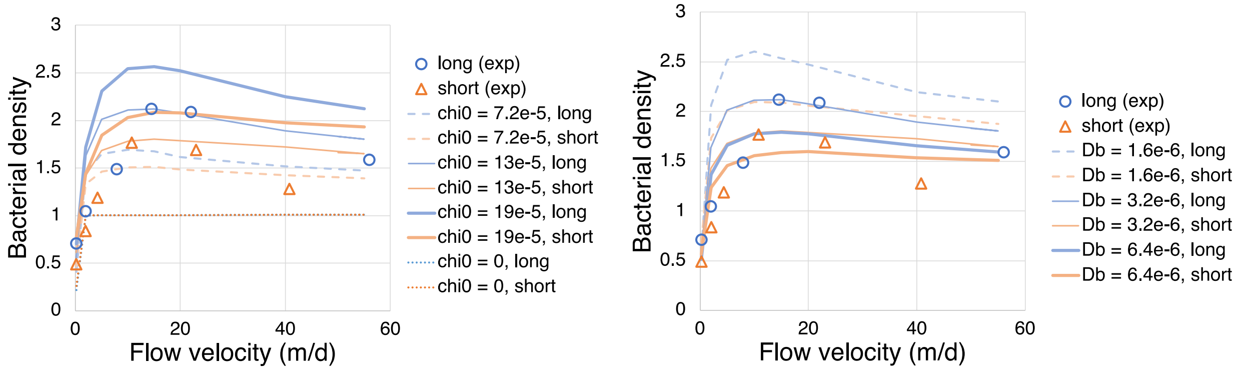

Figure S2. Simulated bacterial densities for various combinations of the diffusion coefficient (D_b) and chemotactic sensitivity coefficient (χ_o).

Bacterial diffusion coefficient D_b and chemotactic sensitivity coefficient χ_o were fitted during the simulations for bacterial distributions, with their ranges informed by literature values. Specifically, D_b ranged from 0.32×10^{-6} cm^2/s[1] to 3.2×10^{-6} cm^2/s [2], while χ_o ranged from 7.2×10^{-5} to 19×10^{-5} cm^2/s [1]. Simulations showed that increasing χ_o or decreasing D_b resulted in higher bacterial densities, as shown in Figure S2. The combination of $D_b = 3.2 \times 10^{-6}$ cm^2/s and $\chi_o = 13 \times 10^{-5}$ cm^2/s provided the best agreement with experimentally obtained bacterial densities. Simulated results in the absence of chemotaxis were also included in Figure S2 to compare them with chemotactic scenarios.

At low velocities (v_f < 5 m/d), the faster attainment of steady state in simulations compared to the experimental results can be attributed to multiple factors. These include flow variability from the syringe pump, the idealized initial condition in simulation with a sharp bacterial front and uniform density, and Tayler dispersion affecting bacterial transport in the triangular region before entering the high-permeability zone in the experimental system. These effects are more pronounced at lower velocities, likely contributing to discrepancies below 5 m/d at 60 min. While it is challenging for the simulations to fully replicate all experimental complexities, the results using $D_b = 3.2 \times 10^{-6}$ cm^2/s and $\chi_o = 13 \times 10^{-5}$ cm^2/s capture key qualitative trends and quantitative agreement within a reasonable parameter range compared to literature values.

Mesh Grid in Simulation

Finer mesh sizes or more mesh grids used in a simulation can solve differential equations more accurately but require higher computation cost. We used the finest mesh grids that our workstation (Dell Precision 5820, 18 cores, 8 GB ram, 128 GB memory) could run as a ground truth and compared errors in bacterial distribution computed at various mesh sizes, as shown in Figure S3a. Finer mesh grids generate more nodes for computation in a finite domain, and computation time increased exponentially as seen in Figure S3b. We applied the mesh grid or number of nodes that are indicated by arrows in Figure S3 in our simulations.

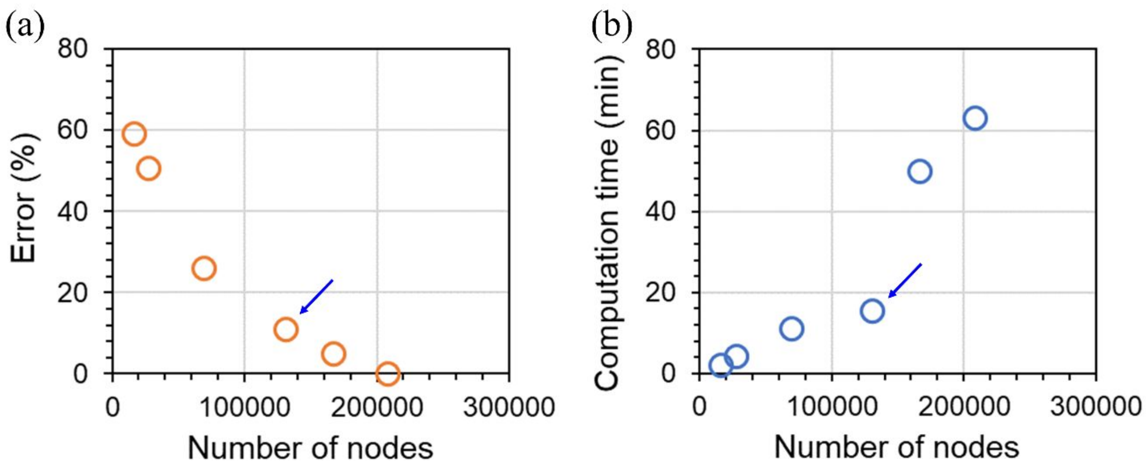

Figure S3. Number of nodes applied in simulations and corresponding computational error estimate (a) and computation time (b).

Shear Rate in Long and Short Micropockets

Bacteria near no-slip walls experience shear forces generated by fluid flow. Figure S4 shows simulated flow velocity in micropockets and shear rates perpendicular to the flow, indicated by the dashed blue arrow at an average inlet velocity of 15 m/d. Bacterial movement has previously been reported to be mediated by shear flow, such as exhibiting Jeffery orbit [3] and rheotaxis [4,5]. Simulations of our experimental system show that shear near NAPL interface was minimal, *e.g.*, less than 1 s^{-1} at 15 m/d as shown in Figure S4b. Thus, the influence of shear on bacterial chemotaxis was considered negligible.

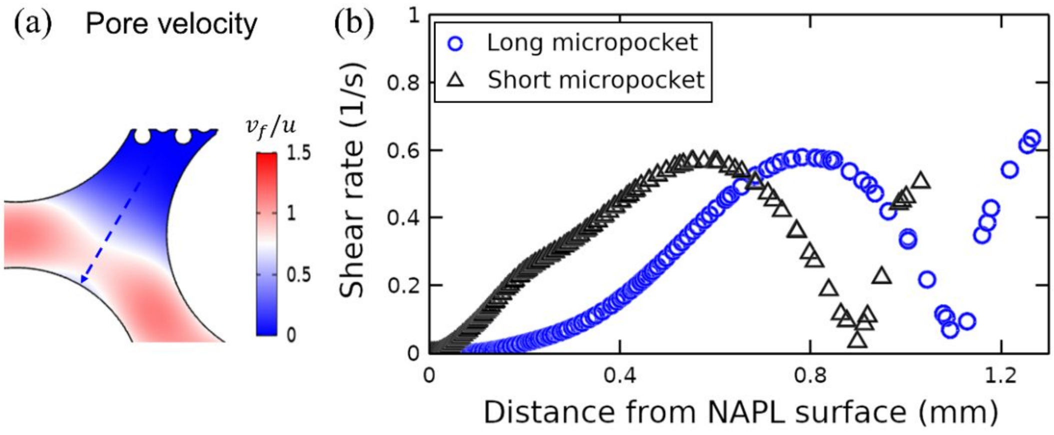

Figure S4. (a) Simulated pore velocity normalized by average fluid velocity at 15 m/d. (b) Shear rate was plotted against the distance from the NAPL-water interface in long and short micropockets. Blue circles represent the shear rate along the dashed blue arrow in (a).

Simulation in Porous Media with Various Dimensions

Our experiments showed that as flow velocity increased, bacterial retention in micropockets due to chemotaxis increased up to an extent and then decreased. To find the correlation among the optimal flow velocity in terms of maximum chemotactic response and potential influential parameters (Figure 3 in the manuscript), we simulated bacterial distributions over a wider range of pore dimensions (d_g, d_p and l), values for which can be found in Figure S5 and Table S2. As illustrated in Figure S5, micropocket locations featured quiescent flow ($v/u <$ 1) and chemical hotspots.

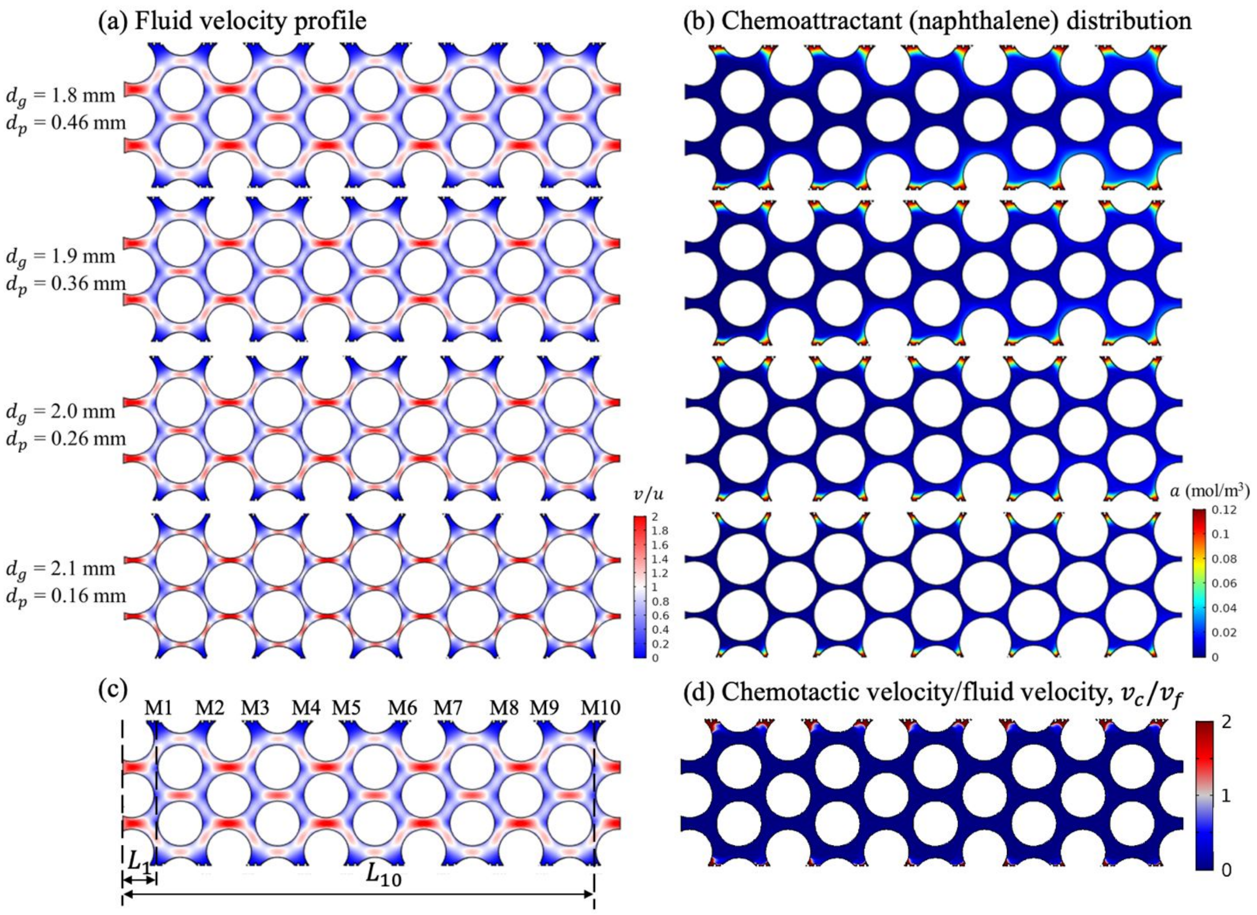

Figure S5. Simulated (a) flow profile and (b) chemoattractant distribution in pore geometries with four different dimensions at an averaged flow velocity of 10 m/d introduced at the inlets on the left. (c) Locations of M1-M10 and their distance L_i from the entrance. (d) A spatial map of v_c/v_f comparing the strength of chemotaxis to convection within micropockets.

Table S2. Pore dimensions and length of micropockets in Figure S5.

Grain diameter d_g (mm)	Pore throat d_p (mm)	Length of micropocket l (mm)
1.8	0.46	1.30 (long)
		1.00 (short)

1.9	0.36	1.25 (long)
		0.95 (short)
2.0	0.26	1.20 (long)
		0.90 (short)
2.1	0.16	1.15 (long)
		0.85 (short)

Simulated Naphthalene Distributions

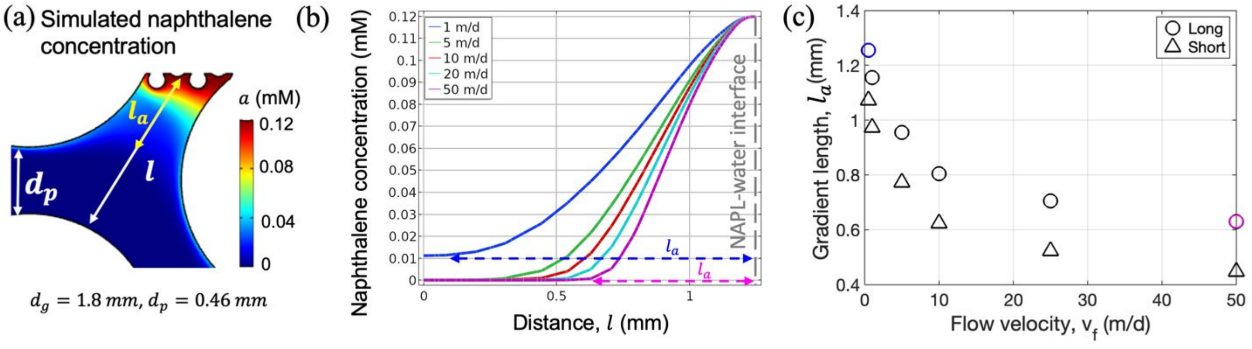

Figure S6. (a) and (b), simulated naphthalene distribution in a long micropocket and naphthalene concentrations along the micropocket length l at varying flow velocities. l_a is the distance from the NAPL-water interface over which a naphthalene gradient exists. (c) Naphthalene gradient length l_a obtained from (b) at each velocity.

Chemoattractant naphthalene partitions from NAPL-water interfaces and diffuses into the surrounding aqueous phase, creating naphthalene gradients (Figure S6a) that vary with different fluid velocities. The distance over which naphthalene diffuses into the aqueous phase, denoted as l_a, indicates the extent of these gradients. As flow velocity increases, steeper naphthalene gradients form, resulting in a shorter gradient length, l_a, as shown in Figure S6b. The l_a value at each

velocity was plotted in Figure S6c with blue and magenta open circles representing gradient distances at 1 m/d and 50 m/d, respectively. The gradient length l_a determines the range of chemotactic response, and therefore we defined the chemotaxis timescale as $\tau_{che} = \frac{{l_a}^2}{\chi_o}$ in the main manuscript.

Estimated Exposure Time to Chemical Gradients from Literature

Chemotaxis has been studied in various models for porous media. We estimated bacterial exposure time to chemical gradients as the ratio of characteristic length (*e.g.*, pore throat) and fluid velocity, and summarized them in Table S3.

Table S3. Chemotactic response and estimated exposure time from published data.

Literature	Chemotaxis (observed)	Characteristic length (mm)	Fluid velocity (m/d)	Exposure time (s)
Long et al. (2009)[6]	yes (strong [a])	0.20	5	3.5
	yes (weak)		10	1.7
	no		20	**0.86**
Wang et al. (2009)[7]	yes	0.45	1.9	20
	yes [b]		5.1	7.6
Wang et al. (2012)[2]	yes (strong)	0.050	0.5	8.6
	yes (weak)		1	4.3
	no		5	**0.86**
	no		10	**0.43**

Roggo et al. (2018)[8]	yes (a_0 varied) [c]	7.8	0.5	1300
Lanning et al. (2008)[9]	yes	6	19	27
Wang et al. (2016)[10]	yes	0.04	0.5	6.9
	no		5	**0.69**
This study	yes	0.46	4	9.9
	yes		8	5.0
	yes		11	3.6
	yes (strong)		15	2.7
	yes (strong)		22	1.8
	no		41	**0.97**
	no		56	**0.71**

[a] Description of 'strong' or 'weak' response was based on comparison of bacterial distributions in the same study.

[b] Two different bacterial species were studied in Wang et al. [7].

[c] Chemoattract concentration (a_0) was varied in experiments of Roggo et al. [8].

Bacterial Residence Time in Micropockets

We revealed the weaker chemotactic response under high flow velocities by varying χ_o values in the simulations, as shown in Figure S7. To achieve a closer alignment with experimental data (Figure S7a), significant reductions in χ_o were required as flow velocity increased, as summarized in Figure S7b.

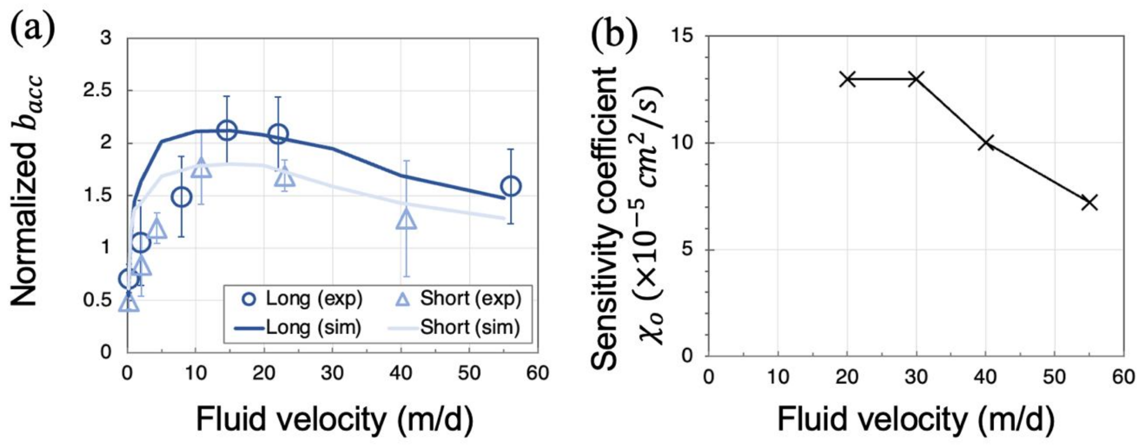

Figure S7. (a) simulated bacterial densities with closer match to experimental data and (b) χ_o values used in simulations for (a).

We calculated the residence time for bacteria entering and exiting the micropockets at velocities of 15 m/d, 40 m/d, and 55 m/d along four streamlines (s1, s2, s3, and s4) as shown in Figure S8a. The residence time was determined as $t = l_s/v_p$, where l_s is the length of the streamline exposed to chemical gradients (the regions between dashed lines in Figure S8b), and v_p is the pore velocity along the streamline. In Figure S8c, residence time decreased for bacteria that were a further distance away from the chemoattractant source. At 40 m/d and 55 m/d, bacteria along streamlines 3 and 4 experience residence times of less than 3 seconds, matching the exposure time $\tau_{exp} = \frac{d_p}{v_f}$ defined in the manuscript.

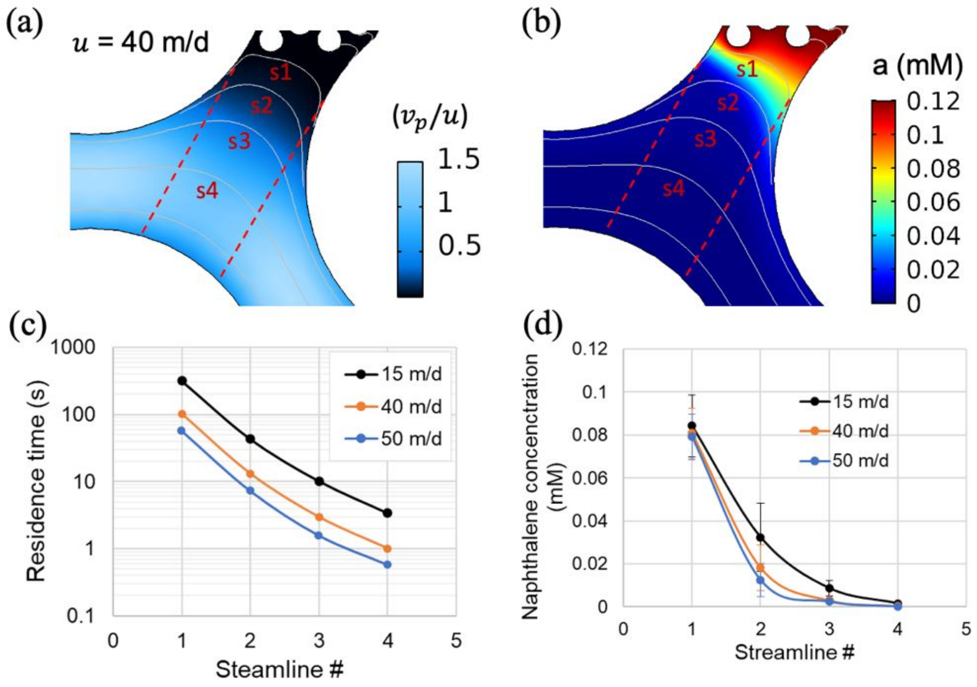

Figure S8. (a) simulated pore velocity v_p and (b) chemoattractant concentration (a, mM) in a long micropocket at average fluid velocity u = 40 m/d in the main flow pathway. (c) residence time $t = l_s/v_p$ and (d) average chemoattractant concentrations along streamlines s1-s4 at three different flow velocities, which are 15 m/d, 40 m/d, and 55 m/d.

REFERENCE

(1) Marx, R. B.; Aitken, M. D. Quantification of Chemotaxis to Naphthalene by Pseudomonas Putida G7. *Appl. Environ. Microbiol.* **1999**, *65* (7), 2847–2852. https://doi.org/10.1128/aem.65.7.2847-2852.1999.

(2) Wang, X.; Long, T.; Ford, R. M. Bacterial Chemotaxis toward a NAPL Source within a Pore-Scale Microfluidic Chamber. *Biotechnol. Bioeng.* **2012**, *109* (7), 1622–1628. https://doi.org/10.1002/bit.24437.

(3) Jeffery, G. B.; A, P. R. S. L. The Motion of Ellipsoidal Particles Immersed in a Viscous

Fluid. *Proc. R. Soc. London. Ser. A, Contain. Pap. a Math. Phys. Character* **1922**, *102* (715), 161–179. https://doi.org/10.1098/rspa.1922.0078.

(4) Wheeler, J. D.; Secchi, E.; Rusconi, R.; Stocker, R. Not Just Going with the Flow : The Effects of Fluid Flow on Bacteria and Plankton. **2019**, 1–25.

(5) Jing, G.; Zöttl, A.; Clément, É.; Lindner, A. Chirality-Induced Bacterial Rheotaxis in Bulk Shear Flows. *Sci. Adv.* **2020**, *6* (28). https://doi.org/10.1126/sciadv.abb2012.

(6) Long, T.; Ford, R. M. Enhanced Transverse Migration of Bacteria by Chemotaxis in a Porous T-Sensor. *Environ. Sci. Technol.* **2009**, *43* (5), 1546–1552. https://doi.org/10.1021/es802558j.

(7) Wang, M.; Ford, R. M. Induced by Chemotaxis in a Packed Column with Structured Physical Heterogeneity. *Environ. Sci. Technol.* **2009**, *43* (15), 5921–5927.

(8) Roggo, C.; Picioreanu, C.; Richard, X.; Mazza, C.; van Lintel, H.; van der Meer, J. R. Quantitative Chemical Biosensing by Bacterial Chemotaxis in Microfluidic Chips. *Environ. Microbiol.* **2018**, *20* (1), 241–258. https://doi.org/10.1111/1462-2920.13982.

(9) Lanning, L. M.; Ford, R. M.; Long, T. Bacterial Chemotaxis Transverse to Axial Flow in a Microfluidic Channel. *Biotechnol. Bioeng.* **2008**, *100* (4), 653–663. https://doi.org/10.1002/bit.21814.

(10) Wang, X.; Lanning, L. M.; Ford, R. M. Enhanced Retention of Chemotactic Bacteria in a Pore Network with Residual NAPL Contamination. *Environ. Sci. Technol.* **2016**, *50* (1), 165–172. https://doi.org/10.1021/acs.est.5b03872.
